# Geographic Variation of Melanisation Patterns in a Hornet Species: Genetic Differences, Climatic Pressures or Aposematic Constraints?

**DOI:** 10.1371/journal.pone.0094162

**Published:** 2014-04-16

**Authors:** Adrien Perrard, Mariangela Arca, Quentin Rome, Franck Muller, Jiangli Tan, Sanjaya Bista, Hari Nugroho, Raymond Baudoin, Michel Baylac, Jean-François Silvain, James M. Carpenter, Claire Villemant

**Affiliations:** 1 UMR 7205 ISYEB, Muséum National d'Histoire Naturelle, Paris, France; 2 Division of Invertebrate Zoology, American Museum of Natural History, New York, New York, United States of America; 3 Unité de Recherche IRD 072-UPR9034 CNRS, Laboratoire Evolution Génomes et Spéciation, Gif-sur-Yvette, France; 4 Université Paris-Sud 11, Orsay, France; 5 School of Life Sciences, Northwest University, Xi'an, Shaanxi, China; 6 Entomology Division, Nepal Agricultural Research Council (NARC), Lalitpur, Nepal; 7 Museum Zoologicum Bogoriense, Indonesian Institute of Sciences, Cibinong, Bogor, Indonesia; 8 CBNBP, Muséum National d'Histoire Naturelle, Paris, France; University of Lausanne, Switzerland

## Abstract

Coloration of stinging insects is often based on contrasted patterns of light and black pigmentations as a warning signal to predators. However, in many social wasp species, geographic variation drastically modifies this signal through melanic polymorphism potentially driven by different selective pressures. To date, surprisingly little is known about the geographic variation of coloration of social wasps in relation to aposematism and melanism and to genetic and developmental constraints. The main objectives of this study are to improve the description of the colour variation within a social wasp species and to determine which factors are driving this variation. Therefore, we explored the evolutionary history of a polymorphic hornet, *Vespa velutina* Lepeletier, 1836, using mitochondrial and microsatellite markers, and we analysed its melanic variation using a colour space based on a description of body parts coloration. We found two main lineages within the species and confirmed the previous synonymy of *V. auraria* Smith, 1852, under *V. velutina*, differing only by the coloration. We also found that the melanic variation of most body parts was positively correlated, with some segments forming potential colour modules. Finally, we showed that the variation of coloration between populations was not related to their molecular, geographic or climatic differences. Our observations suggest that the coloration patterns of hornets and their geographic variations are determined by genes with an influence of developmental constraints. Our results also highlight that *Vespa velutina* populations have experienced several convergent evolutions of the coloration, more likely influenced by constraints on aposematism and Müllerian mimicry than by abiotic pressures on melanism.

## Introduction

Geographic variation of coloration is one of the most striking aspects of diversity within many species [1], [2]. Numerous factors could influence this colour variation, including evolutionary drift [3], but also various selection forces triggered by environmental differences between populations [1], [4]. One of the factors of selection on coloration is aposematism, the use of a highly visible signal to warn predators of unpalatability [4]–[6], such as the black and light stripes of wasps [7]–[9]. Efficiency of aposematic signals relies on the frequency of this signal in the habitats and its probability to be experienced by predators [4], [10], [11]. Therefore, it may seem counter-intuitive to find colour diversity under aposematic constraints. However, colour variation occurs in many aposematic species [11]–[13]. In these species, selection on aposematic signals can also interact with other selective pressures for driving the colour variation in the species. For example, the main colour variation within wasp species is a change in the degree of dark pigmentation, or melanisation, inducing light- or dark-coloured morphs and confusing the taxonomy of the group [14], [15], [16]. This melanism is known to be of adaptive importance in insects: it has been related to crypsis, to thermoregulation and to the resistance to pathogens [17]–[20]. Selection on melanism can thus interact with selection on aposematic patterns of coloration [21]. Surprisingly, the potential relations of intra-specific colour variation with aposematism and melanism have been rarely studied in social wasps (but see [16]). In this study, our aim is to determine which factors influenced the geographic variation of coloration within a species of wasp.

In many organisms, melanism is expressed by a roughly homogeneous increase of the melanin pigmentation over the body [19], [20]. On the contrary, in aposematic insects such as wasps and ladybirds, contrasted colours are a major component of the warning signal. In these organisms, melanism occurs through an increase of the area of delimited melanic patterns on the body [22], [23], [19]. We thus described the variation in coloration of the species by the variation of these melanic patterns.

Warning signal in wasps includes the colour patterns of the different body segments. In order to maintain a recognizable warning signal among the different colour variants, the variation of body parts may follow an integrated process over the entire body, *i.e.* the different segments would vary in a coordinated way, thus maintaining the unity of the general pattern [24], [25]. Furthermore, the different body parts are not likely to play an equivalent role in the warning signal. The coloration of some body parts may be either affected by stronger selective pressures or be under the influence of the same developmental pathways. These phenomena would result in modularity [26], *i.e.* some body parts co-varying more between them than with the other parts, forming colour “modules”. Integration and modularity may enhance the quality of the warning signal of the different colour variants by conserving a coherent pattern between the different body parts involved in this signal.

The evolutionary history of the species is also a major component in understanding the colour variation. In case of highly convergent variation in distantly related populations, the melanism variation could be induced by selective pressures caused either by abiotic factors related to climate, or biotic factors related to aposematism. If melanism convergence was caused by abiotic factors, melanisation should present geographical or altitudinal clines related to climatic proximity [27]. On the other hand, the geographical variation of coloration driven by aposematic pressures would more likely reflect a mosaic of locally selected phenotypes than a cline [28].

**Figure 1:**
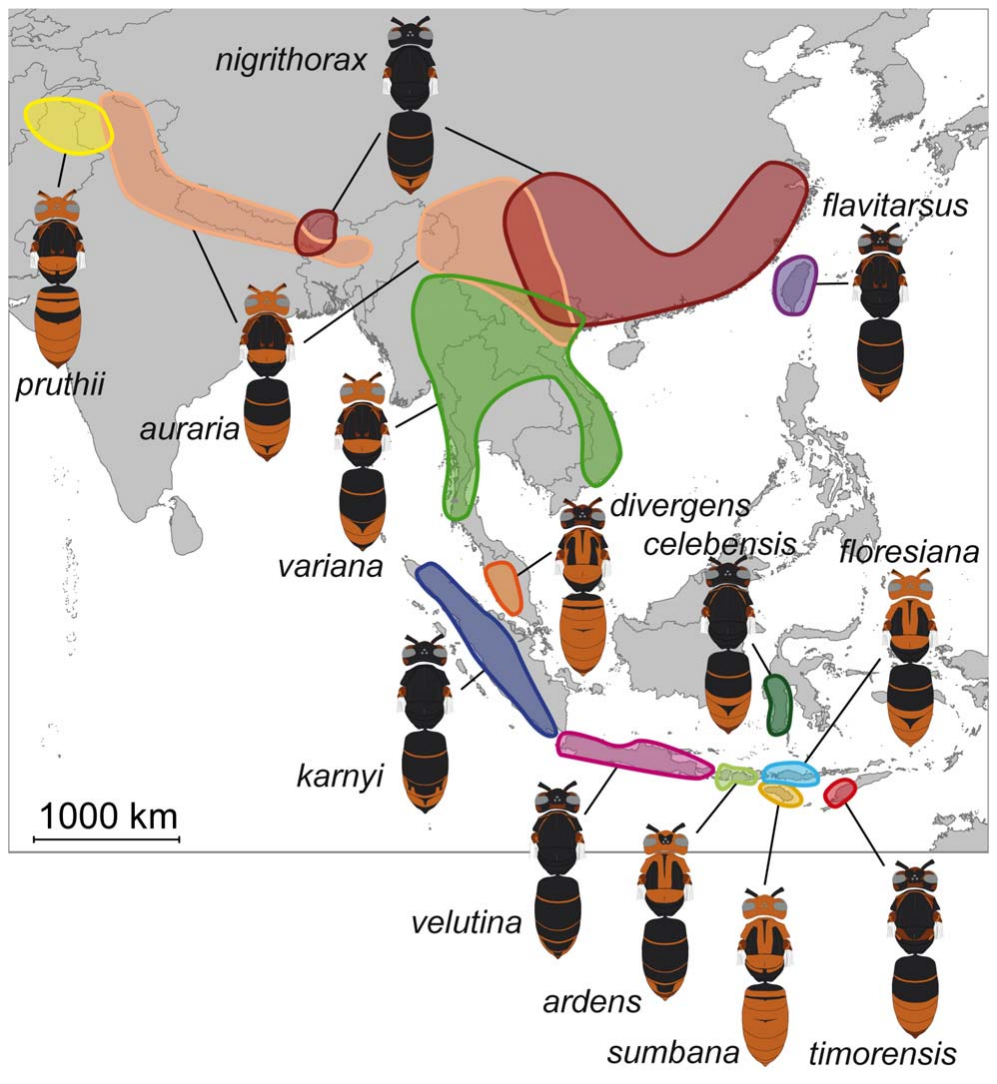
Known distribution of the different colour morphs of *Vespa velutina* across south-east Asia.

We studied the colour variation of the yellow-legged hornet *Vespa velutina* Lepeletier, 1836 which presents a dozen distinct colour morphs across its distribution in South-East Asia [15], [22], [29] (Fig. 1). Its coloration varies among populations from almost entirely yellow or orange to extensively black [15], [30], and can be labile within populations. This phenomenon underlay previous distinction of a second species, *Vespa auraria* Smith, 1852, because of the apparent sympatry in different localities of this colour form with another form called *nigrithorax* [14], [31].

In order to assess the evolutionary history of the species across its distribution and to disentangle potential taxonomic differences that could bias the analysis, we first explored the genetic relatedness of *Vespa velutina* populations using mitochondrial DNA sequences and microsatellite markers. Then, to quantify the colour variation of the individuals, we built a “colour space” from the gradual colour variation between populations based on the measure of melanic patterns of the different body parts. We tested the integration of the melanic variation and its modular nature through the correlation of the body parts vectors in the colour space. Finally, we tested whether the colour variation across the species was congruent with the genetic variation, geographic clines or a spatial mosaic by comparing geographic, genetic and phenotypic distances. These analyses allowed us to answer the following questions:

1. Is *auraria* a lineage genetically divergent from the other colour morphs of *V. velutina*?
2. Is the colour variation of hornet well depicted by a “colour space” based on melanic patterns?
3. Was the coloration of an aposematic species influenced by developmental processes such as integration and modularity?
4. Was the colour variation driven by genetic variation or differences in climatic niches?

We then discussed the implications of our results for the understanding of the development and variation of colour patterns in social wasps.

## Material and Methods

A total of 448 specimens of *Vespa velutina* from 216 localities were studied, including 125 recently collected specimens from 69 different localities from Nepal, China, Laos, Thailand, Vietnam and six Indonesian islands. Other specimens used in this study were assembled from public and private collections. No endangered or protected species were sampled for this study. Specimens sampled in Indonesian and Vietnam were collected partially in national parks with the corresponding authorizations: the research permits were obtained from the head office of the forest protection and nature conservation in Indonesia and from the Tropical Institute for Biology in Vietnam; the permits to collect in National Parks (NP) were delivered by the local authorities of Gunung Rinjani NP in Lombok (Indonesia), of Laiwangi Wanggameti NP in Sumba (Indonesia) and of the Bidoup-Nui Ba NP in Vietnam. The localities sampled in other countries were not protected in any way. No specific permits were required for these collections made in collaboration with local researchers and authorizations of the land owners. Indonesian vouchers were stored at the Museum Zoologicum Bogoriense. Other vouchers were housed in the Museum National d'Histoire Naturelle in Paris. The study of public collection specimens was allowed by curators Y. Gérard and A. Drumont (Institut Royal des Sciences Naturelles, Brussels), J. van Achterberg (Nationaal Natuurhistorisch Museum Naturalis, Leiden), G. Broad (Natural History Museum, London) and F. Gusenleitner (Oberösterreichischen Landesmuseen, Linz). The study of private collection specimens was granted by their owners J. Gusenleitner, J. Haxaire, J.-L. Renesson and P. Tripotin.

**Figure 2:**
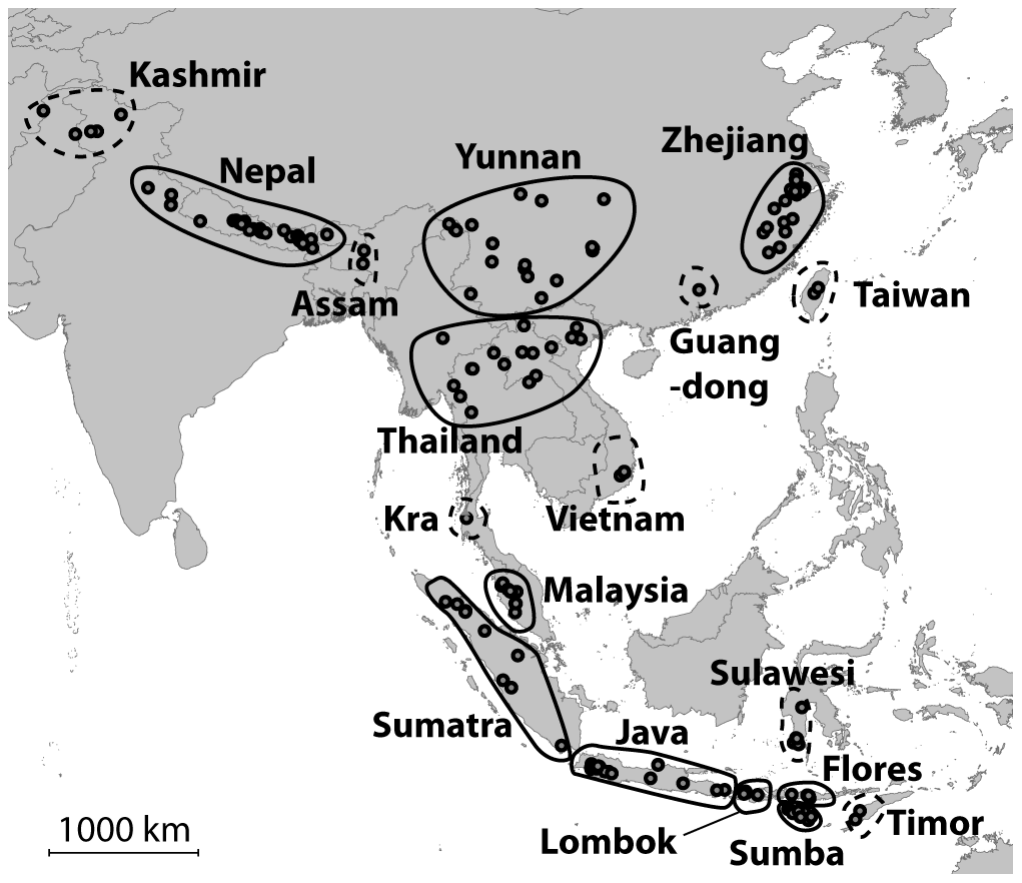
Sampling of *Vespa velutina* across the distribution of the species. Dotted populations are represented by less than 10 specimens.

Over the 216 studied localities, populations were delineated based on colour morphs, geographic distances and ecological environments. We defined ten continental populations (Kashmir, Nepal, Yunnan, Zhejiang, Guangdong, north of Vietnam, south of Vietnam, Thailand, Kra and Malaysia), and eight insular populations from Taiwan and nine Indonesian islands (Sumatra, Java with Bali, Lombok with Sumbawa, Flores, Sumba, Sulawesi and Timor) (Fig. 2, Table 1). Four populations (Kashmir, Nepal, Thailand and Yunnan) each encompass two to three colour morphs for which colour distributions are known to overlap in these areas (Fig. 1). The delineation of these populations was based on the geographic distances of collected individuals and altitude. For example Yunnan specimens were defined as specimens from the mountains while Thailand specimens were defined as located at lower altitude in the North Indo-Burman valley. Three closely-related species were used as outgroup: *V. bicolor* Fabricius, 1787, *V. simillima* Smith, 1868, and *V. vivax* Smith, 1870 [32].

**Table 1:** Population sampling.

Name	Distribution	Colour form	Sampling	Molecular
Kashmir	Kashmir, India, Afghanistan	*auraria, pruthii*	9	0/0
Nepal	Nepal, India, Bhutan	*auraria, nigrithorax*	68	29/31
Assam	India	*auraria*	3	0/0
Yunnan	Bhutan, China (Yunnan), Myanmar	*auraria, nigrithorax*	33	13/15
Guangdong	China (Guangdong)	*nigrithorax*	2	0/0
Zhejiang	China (Zhejiang, Shanghai, Jiangsu, Jiangxi, Fujian)	*nigrithorax*	63	29/23
Taiwan	Taiwan	*flavitarsus*	2	0/0
Thailand	Myanmar, Thailand, Laos, north of Vietnam	*auraria, variana*	34	2/2
Vietnam	South of Vietnam	*variana*	10	7/8
Kra	Thailand (Kra Isthmus)	*variana*	1	0/0
Malaysia	Malaysia	*divergens*	34	0/0
Sumatra	Indonesia (Sumatra)	*karnyi*	25	0/0
Java	Indonesia (Java, Bali)	*velutina*	54	4/4
Lombok	Indonesia (Lombok, Sumbawa)	*ardens*	40	5/4
Flores	Indonesia (Flores)	*floresiana*	25	4/5
Sumba	Indonesia (Sumba)	*sumbana*	27	0/1
Sulawesi	Indonesia (Sulawesi)	*celebensis*	10	2/3
Timor	Indonesia (Timor)	*timorensis*	8	0/0

Sampling column refers to number of specimens studied for the coloration and molecular column refers to specimens that provided CO1 / microsatellite data.

### DNA extraction, Polymerase Chain Reaction, Sequencing and Genotyping

Recently collected specimens were preserved in 95% ethanol. Genomic DNA was extracted from legs using QIAGEN ‘DNeasy tissue Kit’.

A 658 bp sequence of the mitochondrial gene cytochrome C oxidase subunit I (CO1) was amplified for 119 specimens of *V. velutina*, one of *V. bicolor*, one of *V. vivax* and one of *V. simillima* using universal primer sequences HCO and LCO [33]. DNA amplification followed the standard Polymerase Chain Reaction (PCR) protocol of the Canadian Centre for DNA Barcoding [34]. PCR products were checked on a 2% agarose gel. The purified PCR products were sequenced in both directions. BIOEDIT 7.0.5.3 and CodonCode Aligner V.3.5 were used to align both strands of DNA [35]. Sequences were truncated to the same length (658 bp) to avoid missing data. No insertions, deletions or stop codons were found in the alignment.

Genotypes of *V. velutina* populations were assessed using 11 of the 15 microsatellite loci previously developed for the analysis of the origin of the invasion of *V. velutina* in France: D2-185, R4-100, R4-114, D3-15, R1-36, R1-75, R1-77, R4-33, R1-137, R1-169 and D2-142 [36], as well as two supplementary loci (List 2015 and List 2020B) [37]. PCR protocols and genotype scoring are detailed by Arca [38]. Because of high frequencies of missing values in the sample caused by low-quality template DNA, the other loci used in Arca *et al.* [36] were not analyzed.

### Genetic analyses

Haplotype network and diversity among CO1 sequences were calculated using NETWORK 4.6.1.0 software [39]. An evolutionary tree based on CO1 sequences was computed using the Maximum Likelihood criterion (ML) under a GTR model with PhyML 2.4.4 [40]. Bootstrap supports were calculated from 1000 resamplings.

We built a Neighbor Joining (NJ) tree [41] of individuals using (microsatellite) shared allele distances (DAS; [42]) with the R software and “ape” library [43], and performed a Principal Coordinate Analysis (PCoA) on these distances. Population structure was explored using Bayesian clustering through STRUCTURE 2.0 software [44]. We used admixture model and correlated frequencies. Length of the burnin and the MCMC were 10.000 and 100.000 respectively. Simulations were iterated 10 times for each number of clusters from K = 2 to K = 15. Specimen assignment to a cluster was defined by the probability of the specimen to belong to this cluster with a threshold of 0.5.

Descriptive statistics of populations based on microsatellite data as the genetic diversity estimate θ were obtained using Arlequin 3.01 [45].

### Colour pattern analyses

The curved nature of most body segments of wasps makes a standardized quantification of colour over the insect body difficult, due to reflectance and luminosity problems caused by the 3D structure. Furthermore, colour of specimens in natural history collections can be altered by the different collecting methods and conservation conditions. Direct colour quantification using common photograph and pixel-based methods are hardly suitable to such analyses. On the other hand, a semi-quantitative characterisation of melanin patterns can be applied.

We identified the melanic patterns, or modalities, of the different body parts using the original descriptions of *V. velutina* colour morphs and the observation of 448 collection specimens. Body parts with constant coloration (*e.g.* clypeus, propodeum), rare colour variation present in less than 5% of the specimens studied (*e.g.* middle- and hind-femora) or variation without clear patterns (*e.g.* coxae) were not included in the analysis.

**Figure 3:**
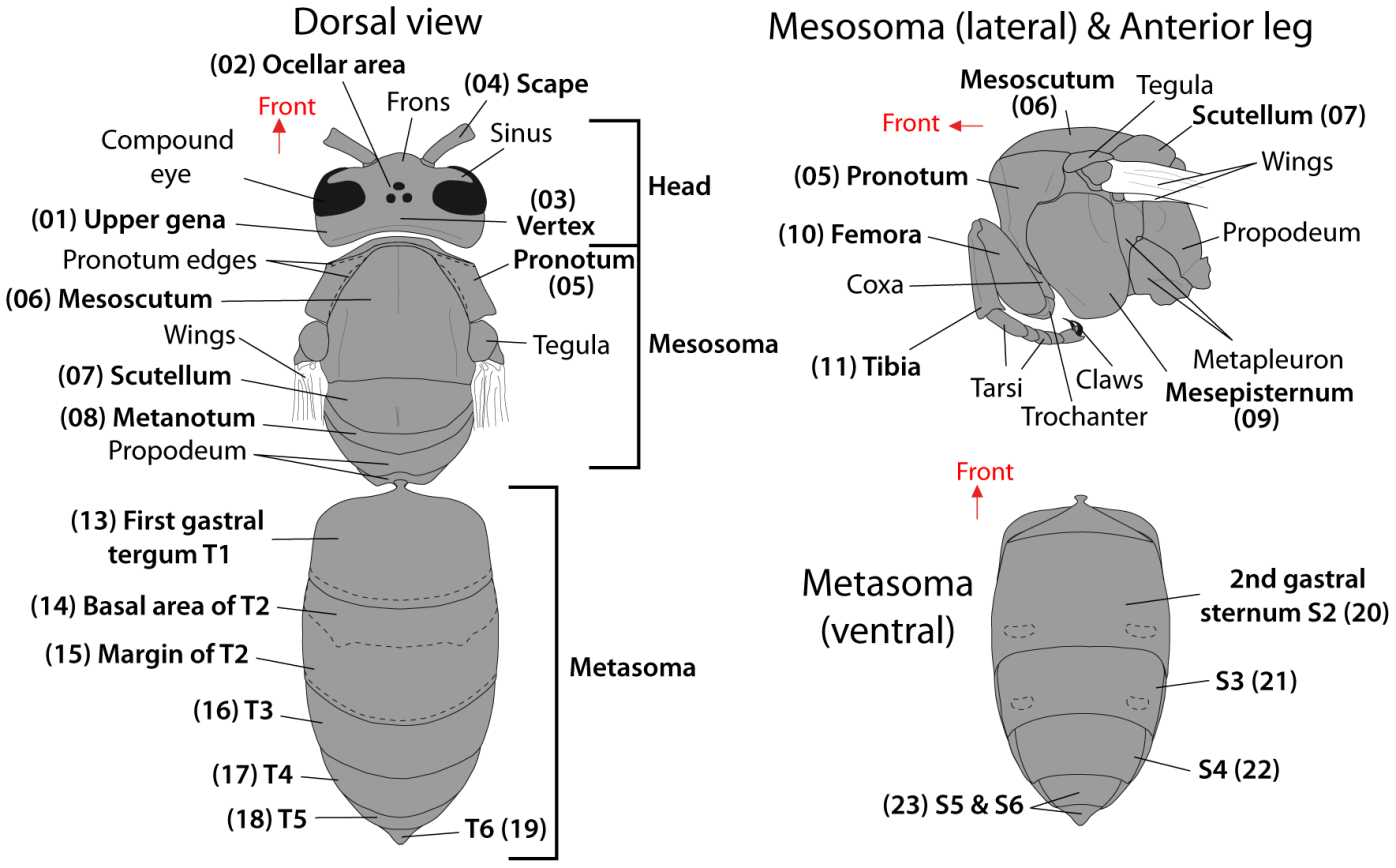
Terminology of the studied body parts of *Vespa velutina*. Characters coding variation of melanisation are in bold and numbered from one to 23. The twelfth character (spot at the apex of the hind-tibia) is not depicted.

**Figure 4:**
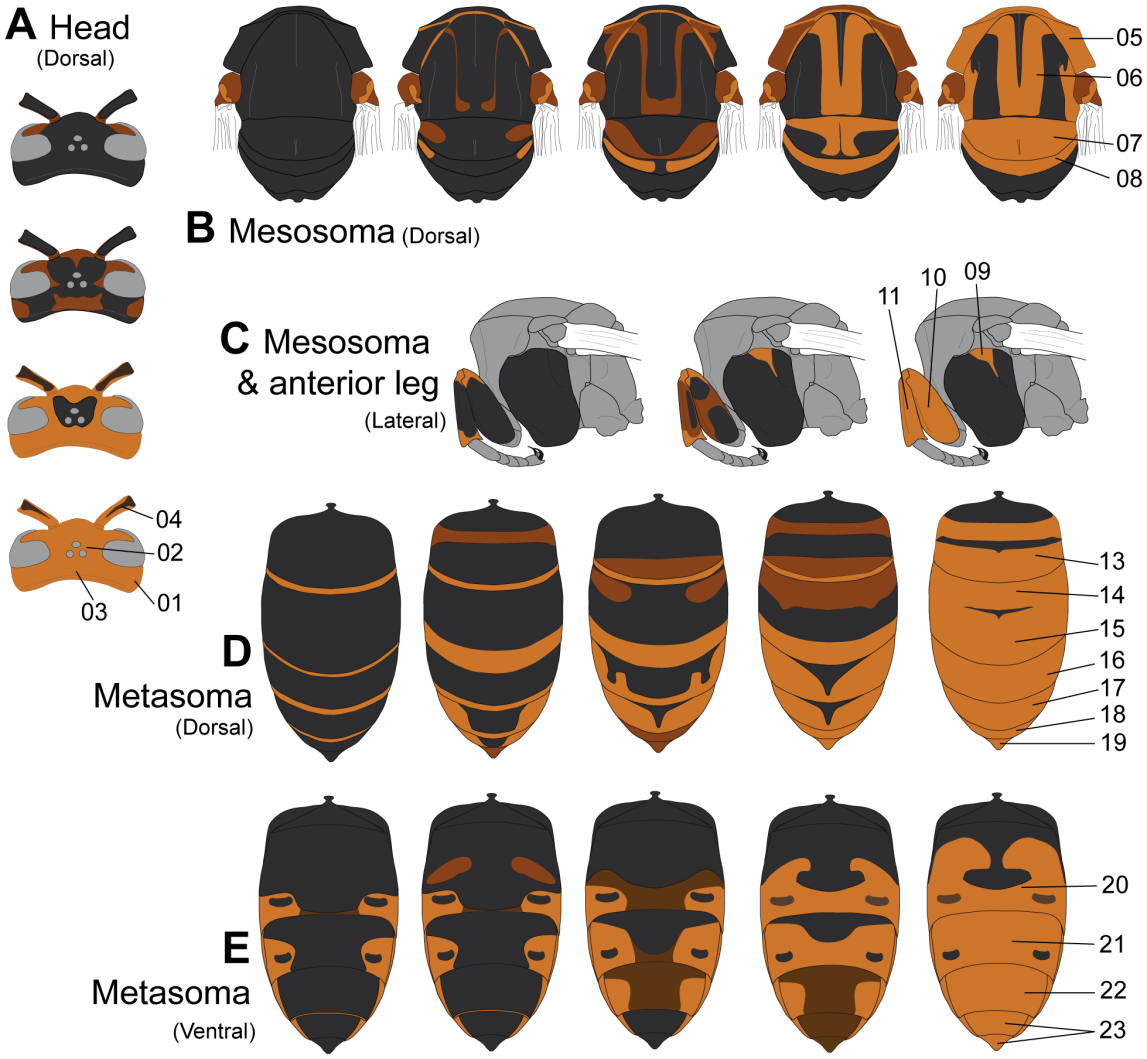
Modalities of colour characters. Each colour character was depicted independently from the others. The ensemble of colour patterns gathered in a given illustration is thus not necessarily reflecting an actual coloration found in wild organisms. **A**. Variation of the four head characters in dorsal view. **B**. Variation of the four dorsal mesosomal characters. **C**. Variation of the lateral mesosoma character and the two anterior leg characters. The hind leg with a spot at the apex of the hind-tibia was not depicted. **D**. Variation of the seven dorsal metasomal characters. **E**. Variation of the four ventral metasomal characters.

We found 23 recurrent colour variations over the body parts, four concerning the head, five the mesosoma, three the legs, seven the metasomal dorsum and four the metasomal sterna (Table 2, Fig. 3). These characters were coded in two to five modalities, giving a total of 73 patterns depicted in Figure 4 (cf. Appendix S1). For each analysed body part, extreme melanic patterns were coded as two binary characters of the presence/absence of the darkest and the lightest modalities. Intermediate modalities were coded following a fuzzy coding between these two extreme characters [46]. In practice, it is equivalent to coding the modalities following their ranking in an ordination of the modalities from one extreme to the other as:
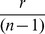
where *r* is the rank of the coded modality, starting from 0 for the extreme modality not described by the character, for example the darkest modality for a character of presence/absence of the lightest modality, and *n* the total number of modalities.

**Table 2:** List and descriptions of colour characters.

N°	Body part	Character	Number of modalities	Illustration (Figure 4)
01	Head	Upper gena	3	A
02	Head	Vertex	3	A
03	Head	Ocellar area	3	A
04	Head	Dorsal margin of scape	3	A
05	Mesosoma	Prothorax	4	B
06	Mesosoma	Mesoscutum	5	B
07	Mesosoma	Scutellum	3	B
08	Mesosoma	Metanotum	4	B
09	Mesosoma	Mesepisternum	2	C
10	Legs	Profemora	3	C
11	Legs	Protibia	3	C
12	Legs	Metatibia apex	2	C
13	Metasoma	1^st^ metasomal tergum	4	D
14	Metasoma	Basal area of the2^nd^ metasomal tergum	2	D
15	Metasoma	Apical margin of the 2^nd^ metasomal tergum	3	D
16	Metasoma	3^rd^ metasomal tergum	4	D
17	Metasoma	4^th^ metasomal tergum	4	D
18	Metasoma	5^th^ metasomal tergum	3	D
19	Metasoma	6^th^ metasomal tergum	2	D
20	Metasoma	2^nd^ metasomal sternum	4	E
21	Metasoma	3^rd^ metasomal sternum	3	E
22	Metasoma	4^th^ metasomal sternum	3	E
23	Metasoma	5^th^ & 6^th^ metasomal sterna	2	E

Characters' location on the organism is illustrated in Figure 3. Modalities of each colour character are depicted in Figure 4.

Only workers were coded in order to avoid bias caused by social colour dimorphism within the species [15].

We analysed the resulting matrix of 46 variables ranging from 0 to 1 using a correspondence analysis (CA). The coding in two opposed variables for each character induced a marginal value identical for every individual, independently from their melanisation level, and so the same weight in the CA. In such analysis, colour variation is estimated by semi-quantitative variables describing the level of melanisation of each body part. Because the modalities of a given body part could also be considered as independent discrete states between populations, we also computed a multiple correspondence analysis (MCA) on a matrix considering each modality as a variable. The second analysis being based on 73 variables, its description of colour variation was seemingly more precise than the one using the fuzzy coding. However, the CA with fuzzy coding has the advantage of describing the variation of melanic patterns of each body part by a linear direction in the resulting multivariate “colour space”. In the MCA, the variation of a single body part is described by a succession of vectors, due to the different modalities.

In order to quantify the accuracy of the colour spaces from the two analyses, we compared the distribution of individuals in these spaces to the classes of colour morphs distinguished by systematists. We tested for the separation of individuals from these different morphs in the colour spaces using cross-validation from a canonical variate analyses.

Both light and dark characters of a body part being vectors with opposite directions from the origin in the CA, we estimated the directions of variation of melanic patterns in the colour space by focusing only on variables describing the lightest modalities. We used these directions to compute a correlation matrix between colour characters in order to explore the potential integration and modularity of the melanisation variation. We tested for melanisation integration in organisms by comparing correlations between characters. If melanisation is an integrated process over the entire body, correlation between “light” variables of the different body parts should be mostly positive.

### Comparison of colour, genetic and geographic diversity

In order to test the congruence of colour pattern variation with genetic and geographic variation, the distances between colour patterns were compared to the geographic, climatic and genetic distances using pairwise vectors correlation (RV) tests on principal coordinates. The RV coefficient is a multivariate equivalent of the correlation coefficient and addresses the relationship between two sets of variables drawn from a same sample [47], [48]. Its significance was tested using an approximation of a permutation distribution with the library “FactoMineR” in R [49], [50]. The different distances were simultaneously available for 84 specimens from eight populations because the genetic sampling comprised fragmented specimens or specimens from the queen caste. Both individual and population distances were analysed. Geographic distances were computed as geodesic distances between GPS coordinates of the different sample localities using the library “oce” [51]. Genetic distances were computed as allele-shared distances and haplotypic distances. Climatic distances were computed on scores of a Principal Component Analysis of eight climatic variables used in a previous niche modelling of the species [52]: Annual mean temperature, Temperature seasonality, Maximum and Minimum annual temperatures, Annual precipitation, Maximum and Minimum monthly precipitations and Precipitation seasonality. These variables were extracted from the BIOCLIM database as five arc-minutes grids (http://www.worldclim.org/; [53]) on the basis of GPS coordinates, and scaled by their standard deviation. Colour pattern variation was described from scores of the CA. Principal coordinates requiring Euclidean distances, genetic and geodesic distances were transformed following Lingoes [54]. Disparity between the distance matrices was visualised using neighbour joining on the dissimilarity matrix with one minus the squared RV coefficient as a dissimilarity index. We illustrated details of the dissimilarity between haplotypic and phenotypic data by plotting the correlation matrix of distances per individuals between the two datasets and their corresponding hierarchical clustering with the “gplots” package [55]. Under the hypothesis of a strong similarity, the structure of the two clustering trees should be equivalent and the correlation of distances for each individual should approach one. Consequently, the correlation matrix of distances should present clear blocks of high and low correlations related to the corresponding clusters.

The low intra-population diversity produced a similar structure in every distance matrix computed on individual distances. In order to test the correlation of phenotypic, geographic and genetic distances among populations, we also computed distances between populations' colour, climatic, geodesic and genetic averages. We used respectively population mean colour and climatic scores, mean GPS coordinates and mean DAS and haplotype distances.

Previous studies used F_st_ and Q_st_ estimates to compare phenotypic and neutral genetic differentiation between populations and to test for geographic clines (*e.g.* [56], [27]). However, these estimates involve the intra-population variation [57] which can hardly be estimated for lowly sampled populations such as some of ours. Furthermore, Q_st_ computation requires an assessment of the heritability and of the additive genetic variance of the phenotype [27], [58]. These values were unknown for the wing shape of social wasps and a sensitivity analysis showed that they critically influenced our Q_st_ estimates. Therefore, we chose not to use these estimates in our study.

## Results

### Haplotype diversity

**Figure 5:**
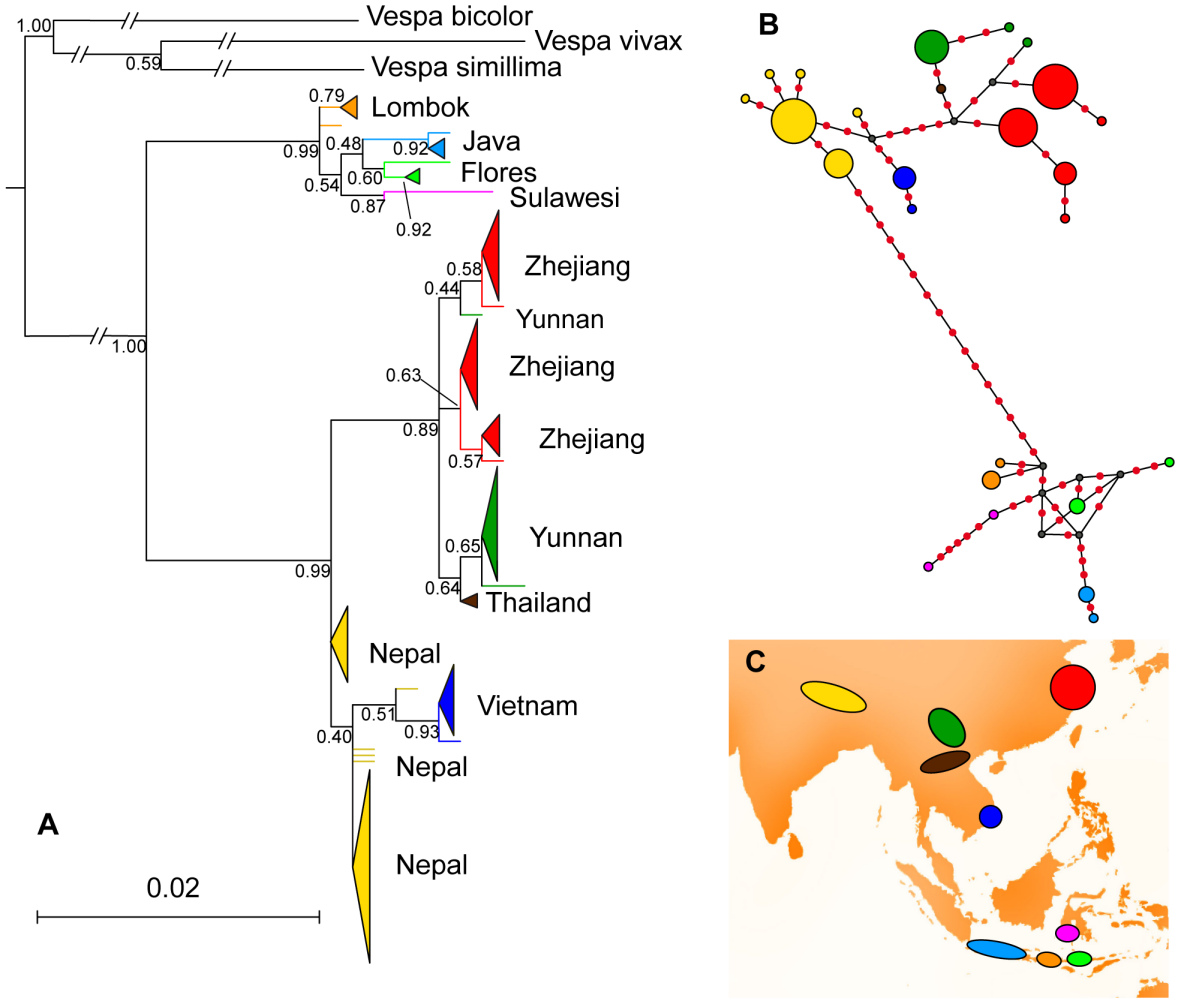
ML tree and haplotype network of CO1 variability of the populations of *V. velutina*. **A** - ML tree computed on CO1 sequences. Scale bar represents the expected mutation per site, node values are bootstrap supports. **B** - Haplotype network. White diamonds are the inferred mutations. **C** - Populations sampled. Size of triangles (A) and circles (C) are proportional to the related number of specimens having these haplotypes.

We found 25 different haplotypes in our mitochondrial DNA sampling of *V. velutina*. Populations were separated into two main clusters: a cluster from the Indonesian archipelago and a cluster from mainland populations (Fig. 5A). More than 15 mutations occurred between these two clusters, while the maximum of divergence within each cluster reaches 11 and 12 mutations for mainland and Indonesian haplotypes respectively (Fig. 5B, C).

In the ML tree Indonesian haplotypes were grouped in the cluster that was the sister group of all other Asian haplotypes. They were more similar to Nepalese haplotypes than to haplotypes from other geographically closer populations. Indonesian haplotypes exhibited high genetic diversity within and between populations without shared haplotypes between islands.

Haplotypes from mainland Asia were split in two groups not entirely congruent with the geography of populations. Nepalese haplotypes were basal or grouped with haplotypes from the southern part of Vietnam but this group was not supported by bootstrap values. Thai and Yunnan haplotypes were related to Zhejiang haplotypes in a relatively well supported group.

### Genotype diversity

Allele-shared distances were congruent with distance computed with CO1 in distinguishing mainland from Indonesian specimens in the NJ tree. Overall, mainland specimens were split between a western cluster with the specimens from Nepal, Thailand and Vietnam, and an eastern cluster with specimens from Zhejiang. Specimens from Yunnan formed three different groups spread over these two clusters.

Nepal and Zhejiang populations displayed on average similar genetic diversity (θ = 2.29 and 2.55 respectively) with similar number of localities sampled, but the highest genetic diversity was found in the Yunnan population (θ = 2.64).

We found a significant departure from Hardy-Weinberg Equilibrium for many loci of the Yunnan population and for the L2015 and the R4-114 loci of the Nepal population. We thus excluded them from the Bayesian clustering.

**Figure 6:**
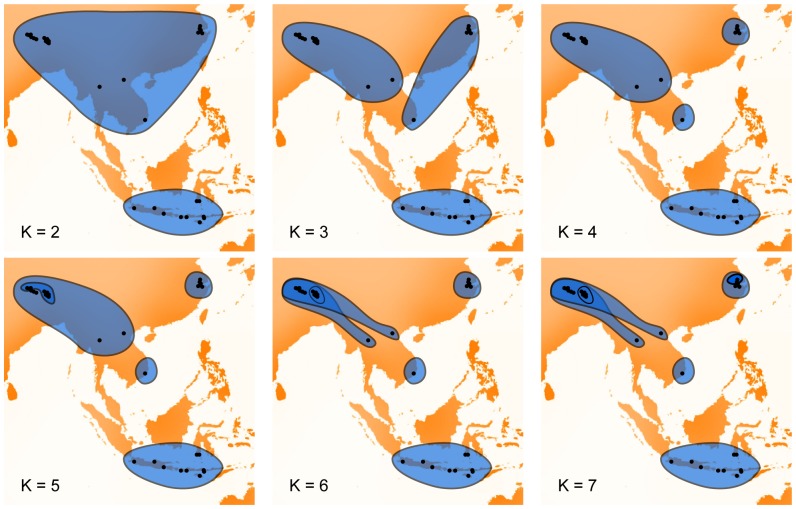
Bayesian clustering of *Vespa velutina* specimens with microsatellite data. Most recurrent results of Bayesian clustering on microsatellite data with increasing number of clusters K. These results were the clusters found in more than 60% of the analyses.

Bayesian clustering distinguished Indonesian and continental populations from K = 2 (Fig. 6). According to Evanno's method, K = 4 would be the best estimate number of clusters in the sampling [59], [60]. At K = 4, continental specimens were split across three clusters, one with Nepal and Thai specimens, a second with Zhejiang specimens and a third with those from Vietnam. With increasing number of K, new clusters occurred mostly within the Zhejiang and Nepal clusters. These relationships may be obscured by the higher allelic diversity found in the Zhejiang and Nepal samples. With K greater than 7, new clusters were not congruent among iterations of simulations. The Indonesian cluster remained as a whole in most iteration, but Sulawesi specimens were found separated from other Indonesian populations in few iterations from K = 5.

### Colour diversity

**Figure 7:**
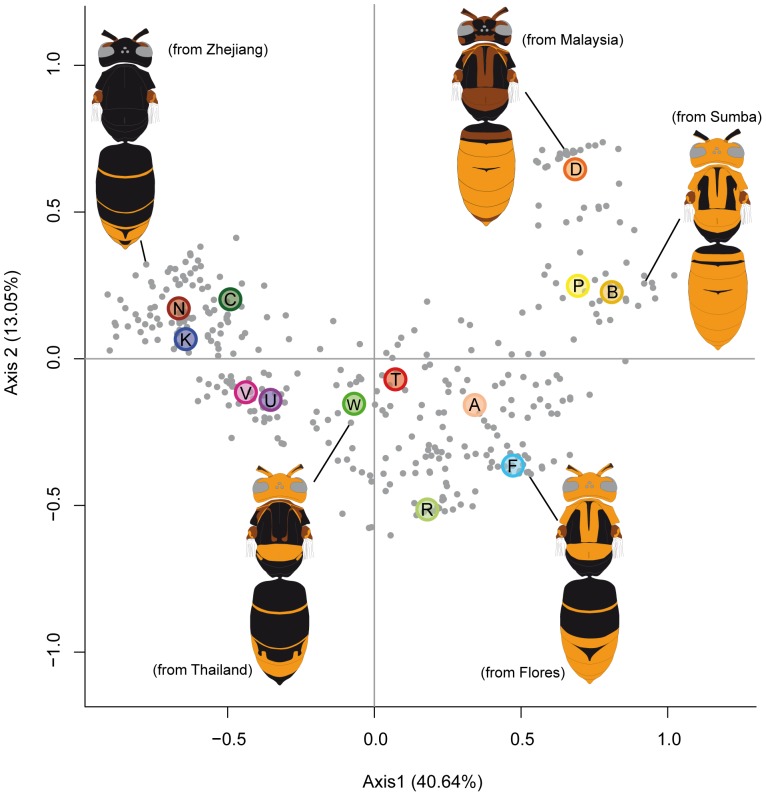
Colour space of *Vespa velutina* specimens. Two first dimensions of the colour space resulting from a correspondence analysis on melanisation described by two binary variables of the extreme light and dark coloration for each body part. Intermediate modalities were integrated using a fuzzy coding. Coloured spots described the mean values of each colour morphs (see Fig. 1). Colour forms: N =  *nigrithorax*. K =  *karnyi*. C =  *celebensis*. V =  *velutina*. U =  *flavitarsus*. W =  *variana*. T =  *timorensis*. R =  *ardens*. A =  *auraria*. F =  *floresiana*. D =  *divergens*. P =  *pruthii*. B =  *sumbana*.

The CA returned a colour space with 23 dimensions of which the first dimension gathered 40.64% of the total variation. This axis described a variation from dark to light characters states (Fig. 7). All the vectors of the lightest modalities were on the positive part of the axis. The second dimension encompassed 13.05% of the variation and essentially opposed the coloration of the metanotum and anterior metasomal dorsum to that of the head, legs and pronotum. The third component, accounting for 9.23% of the variation, opposed the coloration of antennal scape and mesepisternum to the coloration of ventral and apico-dorsal metasomal surfaces and the presence of a spot on hind-tibia. The fourth to seventh components mostly described the variation of two characters in relation to all the others: the spot at the apex of the hind-tibia and the spot on the mesepisternum.

Cross-validation confirmed that the colour space separated most of the colour morphs: more than 92.05% of specimens were correctly attributed to their respective colour morph. Of the 31 specimens misidentified from their colour coding, six corresponded to bad discrimination between the *auraria* and *pruthii* morphs, for which the main divergence concerns leg colours not taken into account in the analysis.

The MCA resulted in a similar distribution of the specimens with the first axis of global melanisation encompassing 20.8% of the variation. This analysis returned a colour-space with higher dimensionality allowing only for a slightly more accurate attribution of the specimens to their respective colour morphs with cross-validation (93.08%). Results of the MCA will thus not be further discussed.

**Figure 8:**
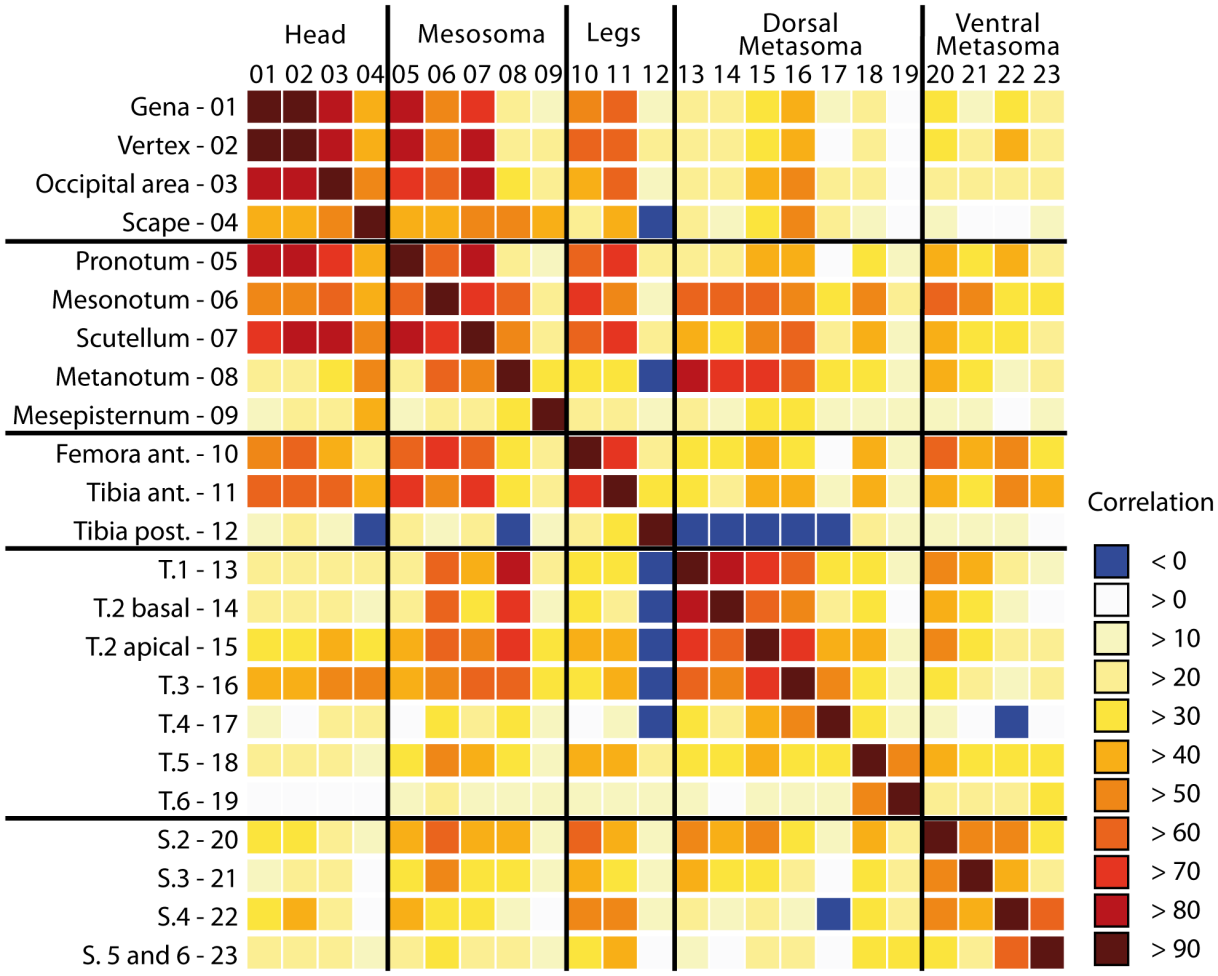
Correlation between the colour variation of body parts. Correlation matrix of the vectors of variation of light colour characters in the colour space (see Fig. 7). Blue marks indicate negative correlation among the two characters.

The correlation matrix computed between the vectors of lighter coloration of the different body parts in the colour space defined by the CA showed 96.84% of positive correlations (Fig. 8). The negative correlations opposed primarily the presence of a colour spot at the apex of hind tibia and the darkening of the fourth metasomal tergum to the darkening of the scape, the metanotum, the first three metasomal terga and the fourth metasomal sternum. Correlation between colour patterns revealed two complexes of correlated characters: one including the three head characters, the anterior leg, pronotum, mesoscutum and scutellum and the other the colour patterns of the metanotum and the three first metasomal terga.

### Comparison of colour, genetic and geographic diversity

**Figure 9:**
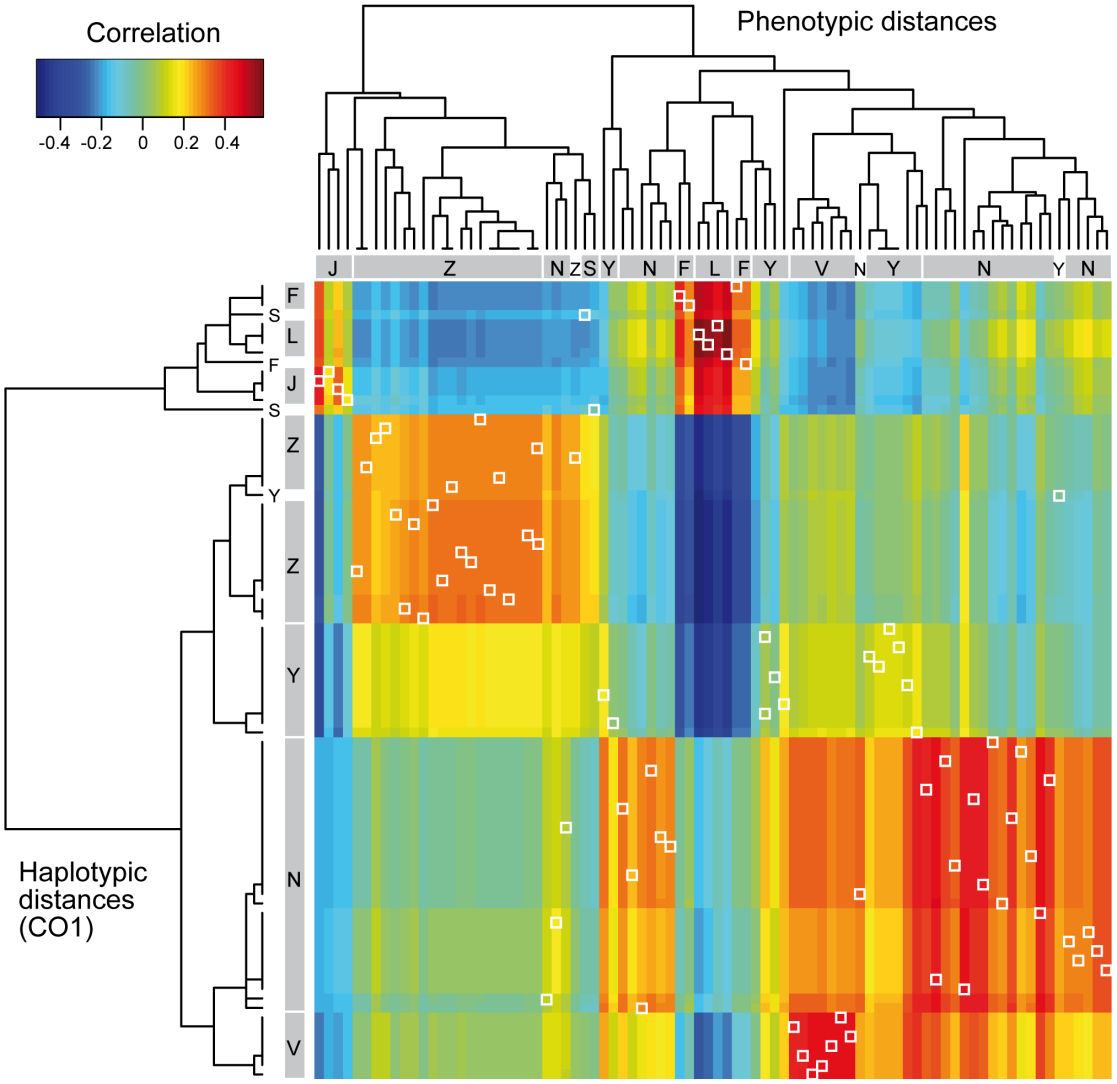
Detailed dissimilarity of haplotypic and colour distances between individuals. Correlation matrix between haplotypic distances (rows) and phenotypic distances (columns) with associated dendrograms. Correlation between distances of a same individual are marked with white squares. Under the hypothesis of similarity between the data, the trees should have the same structure and the individual correlations should approach one. Furthermore, high correlations should be organised in well delimited blocks corresponding to clusters. Haplotypic distances were computed on CO1 sequences and phenotypic distances were computed as the Euclidean distance between individuals in the colour space. Correlation coefficients ranged from −0.6 to 0.6. Dendrograms resulted from complete-linkage clusterings and should not be interpreted as evolutionary trees. Order of specimens differs in rows and columns. Populations: L =  Lombok; F =  Flores; J =  Java; N =  Nepal; S =  Sulawesi; V =  Vietnam; Y =  Yunnan; Z =  Zhejiang.

Comparisons using RV test on the individual distances resulted in a significant relatedness between every dataset. The RV coefficient was the highest between climatic and geodesic distances (RV = 0.680) and the lowest between mitochondrial and colour distances (RV = 0.163). Correlation matrix and hierarchical clustering showed low correlations and clear differences in the structure of these haplotypic and phenotypic distances (Fig. 9). Correlation between the two types of distances was low for most of the specimens. Overall the correlation matrix was not highly structured. Clustering using mitochondrial distances grouped individuals per populations except for Sulawesi, Flores and one specimen from Yunnan, and distinctly separated continental and Indonesian populations. On the contrary, phenotypic distances split most populations and mixed distant specimens. The two main colour clusters separated specimens from Zhejiang, Java, Sulawesi and some specimens from Nepal in one group characterized by a dark head and mesosoma, and specimens from Yunnan, Zhejiang, Vietnam, Flores and Lombok populations presenting a lighter head and mesosoma, in a second group.

Population distances returned fewer significant relationships between the different datasets. Mitochondrial, geodesic and climatic distances were still significantly related but these datasets were no more related to the colour distances and to distances based on genotype data (Table 3).

**Table 3:** Results of pairwise RV tests between populations.

	Haplotype (CO1)	Genotype (Das)	Geodesic distances	Climatic dissimilarity	Coloration dissimilarity
Haplotype (CO1)	-	*0.53090*	0.00882*	0.00456*	*0.63067*
Genotype (Das)	0.390	-	*0.61353*	*0.51280*	*0.62455*
Geodesic distances	**0.824**	0.387	-	0.00586*	*0.69145*
Climatic dissimilarity	**0.900**	0.479	**0.827**	-	*0.58485*
Colour dissimilarity	0.192	0.533	0.181	0.267	-

RV tests were applied on principal coordinates of genotypic, haplotypic, geodesic, climatic and coloration distances between populations. RV coefficients are in the lower triangle of the table with significant relationship in bold, *P* of the tests are in italic in the upper triangle.

Significance: “*” <0.05.

## Discussion

### Evolution of *Vespa velutina*

Our results highlighted two main genetic groups of populations: one from continental Asia and the other from the Indonesian islands. Genetic variation for CO1 within the Indonesian cluster was equivalent to the variation observed within the continental group. However, the structure of the Indonesian variation was not congruent with geography. The low sampling of these islands limited a more detailed interpretation of this structure and may explain the absence of resolution found with the Bayesian clustering on microsatellite markers. Within the continental cluster, mitochondrial and nuclear markers returned distinct results. Sequences of CO1 clustered populations from Nepal and Vietnam while Thailand, Yunnan and Zhejiang specimens were in a different clade (Fig. 5). The microsatellite markers supported a different partition of populations with Nepal and Thailand as a group, Zhejiang and Vietnam being independent populations (Fig. 6). These differences could be explained by the different transmission of mitochondrial and nuclear DNA combined with the unequal sample sizes of populations. Bayesian clustering returned subgroups within the two populations with the largest samples, Nepal and Zhejiang (Fig. 4), while the discrepancies in results between markers involved Vietnam and Thailand populations, both having very low sample diversity. Subgroups found within the continental group should therefore not be considered as relevant biological groups without further genetic data. Both markers confirmed nonetheless that Yunnan sample was genetically heterogeneous, potentially influenced by high migration from the surrounding populations.

The strong genetic difference between continental and Indonesian populations suggested an ancient divergence of these groups of populations. It also raised the question of a potential speciation of southern populations. Two populations from Sumatra and Peninsular Malaysia are located between these continental and southern populations. The absence of molecular data from these two intermediate populations limits our understanding of the isolation of the Indonesian and continental populations. Furthermore, the lack of morphological characters discriminating the two main groups did not support the hypothesis of a long isolation leading to a speciation event.

The presence of a continental cluster confirmed that populations of the *auraria* colour form belong to the same species as populations of the *nigrithorax* and *variana* forms [31]. Populations of the *auraria* colour form were long considered as a different species on the basis of sympatry with the colour form *nigrithorax* in north-eastern India, Nepal, Burma, Assam and western China [29]. In fact, these two colour forms may interbreed: they present intermediate coloration forms in a single locality, as in our Nepal sample presenting typical *auraria* specimens and darker specimens with a coloration somewhat similar to the *nigrithorax* form as observed in northern Vietnam [31]. Considering the close relationships between Nepalese *nigrithorax* and *auraria* specimens, the different populations of *nigrithorax*, and possibly *auraria*, observed in Figure 1 are likely convergent evolution in different populations.

### Patterns of melanisation

We quantified the variation of melanism across *V. velutina* distribution by decomposing the global melanisation pattern into a suite of discrete variations of the different body parts. Some of the colour variation between the populations of *V. velutina* could not be included in this analysis focusing on defined melanic patterns. For example, the differences in light colour that are clearly yellow or reddish in the different populations were not coded and some variation in leg melanisation was too labile or too rare to be taken into account without overweighting these characters. However, the high rate of correct identification of specimens to their colour morph confirmed that a characterisation of melanin patterns of the different sclerites is a good estimate of the colour variation across the species. Furthermore, the use of a fuzzy coding forcing each character variation to follow a linear direction in the colour space minimally affected the results, when compared to a more complex colour space of each variation considered as a discrete binary variable.

This quantification of melanism highlighted that the melanisation of numerous sclerites varies differently in *V. velutina* (Table 2). Each sclerite tended to have a well defined set of particular and complex melanin patterns across the distribution (Fig. 4). These patterns involved mostly an antero-posterior extension of the black stripes, on the metasoma (Fig. 4.D and E). Other segments like mesoscutum, metanotum or mesepisternum presented well-defined marks of light colours which size may vary (Fig. 4.B). Finally, these patterns seemed restricted to a given sclerite, not extending to different segments.

### Genetic bases of melanin production and patterning genes

The genetic control of pigmentation has been widely studied, notably in fruit flies and butterfly wings [61]–[64]. This genetic control occurs at different scales in melanin synthesis: directly on the genes coding for proteins of the synthesis chain, like the *yellow* gene [61], [62]; indirectly on genes coding for proteins altering melanin precursors, like *ebony* [65]; or on genes modifying the expression of these two previous groups of genes, like *engrailed* or *Abdomen-B* [66]. Patterning expression of these genes has been related to the diffusion of small compounds called “morphogens” from source cells through the tissues during the development [67]–[69]. Morphogen diffusion is tightly related to temperature, but also to body topology: for example, wing veins act as barriers in signal diffusion across the wing [70], [71]. This may explain why some light or dark spots in hornets do not extend to their neighbouring sclerites through cuticular sutures, such as those on the metanotum or mesepisternum (Fig. 4).

**Figure 10:**
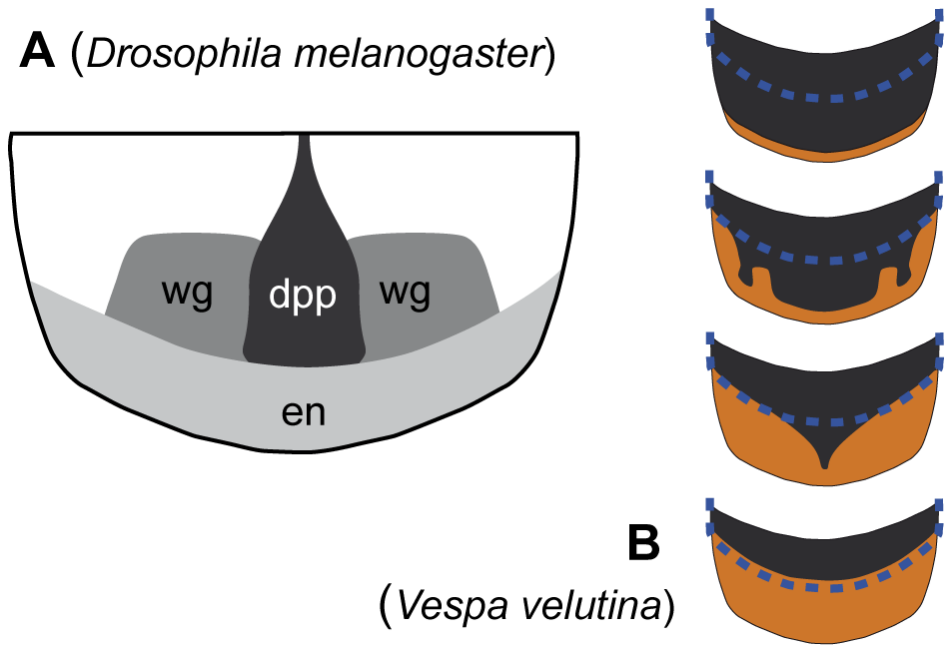
Expression patterns of regulatory genes and variation of melanisation in metasomal segments. **A**. Expression patterns of *engrailed* (en), *wingless* (wg) and *decapentaplegic* (dpp) on an abdominal tergum of *Drosophila* (modified from [75], after [72], [74]). **B**. Variation of melanisation of the third metasomal tergum of *V. velutina*. The blue dotted lines represent the part of the segment covered by the second tergum.

In our analysis, the black and yellow stripes of the different metasomal segments were formed by an apical yellow stripe extending more or less anteriorly and often interrupted by a median band and sometimes lateral spots of melanin (Fig. 4.D). This variation appeared strikingly similar to the patterned activities of three genes Hox, *decapentaplegic*, *wingless* and *engrailed*, regulating the abdominal pigmentation in *Drosophila melanogaster* [72]–[74] (Fig. 10). In the fruitfly, *engrailed* was found responsible for the light band on the posterior margin by repressing the expression of the *yellow* gene, blocking the production of melanin [74]. On another hand, *decapentaplegic* seems to enhance the formation of the median band of melanin [72]. Interaction between these three genes and the variation of their level of expression could explain the complex patterns of melanisation of the metasomal segments of social wasps [75]. These three genes are probably not the only genes involved in the variation of melanin patterns, but they may be good candidates for identifying genes involved in the formation of patterns in metasoma melanisation.

### Variation of melanisation between the different segments

The first axis of variation in the colour space described a global variation in melanisation and appeared positively correlated to the 23 variables of light coloration (Fig. 7). Furthermore these variables were positively correlated in 96.84% of the pairwise comparisons. The correlation between vectors of melanisation of each body part was not homogeneous and suggested two modules: one combining the melanisation of the cephalic capsule together with the pronotum and scutellum, and another the melanisation of the three first metasomal terga with metanotum and in a lesser extent with mesoscutum (Fig. 8). Together, these results suggested a partial integration of melanisation variation over the body, with potential regionalisation of this phenotype.

Because the aposematic signal is located over the entire body in social wasps, such integration and modularity may enhance the quality of the warning signal of the different variants. Colour integration over the body may result from variation of the general regulation of coloration. Two regulation processes of the pigmentation are hormonal levels, melanin production being related to ecdysone titre [75], [76], and genetic variations. Sensitivity of genes involved in melanin synthesis to morphogens may differ from one population to another: the influence of the different morphogens in inhibiting or stimulating a gene expression results from the ability of their transcriptional regulators to bind to a regulatory region of the DNA next to the coding part of the gene [77]. For the *yellow* gene, a *cis-*regulatory promoter has been identified as responsible for pigmentation difference between species due to evolutionary changes altering the number of binding sites of the regulators [78], [66]. Variation in these *cis-*regulatory sequences returned different pigmentations of several body parts, either the whole abdomen, sometime with the thorax, or only the two last abdominal segments. The integration of melanism over the body and its structure in modules may thus be linked to variation in hormonal production, but also to a difference in regulatory genes' sensitivity to morphogens' signals that evolutionary changes alter from one population to another.

### Colour variation between populations

Our results of comparisons of distance matrices showed that colour pattern diversity did not match the evolutionary history described by our analysis of molecular markers, nor the geographic distances or the climatic similarity between populations (Figs. 9). Climate, geographic and mitochondrial based distances presented similar structure, but the data based on genotypic and phenotypic distances were differently structured. The absence of congruence between genotypic and CO1 sequences or geography may indicate an influence of the restricted population sampling on the genotypic distances. Another hypothesis would be an unbalanced dispersal of males and females in the species, with males dispersing more than females; however this is not congruent with our current knowledge of this species [79], [52].

Non congruence of colour-based distances with the other dataset was explained by the presence of several populations having very dark colour patterns both in Indonesia and on the continent, while a southern Indonesian population has the lightest colour morph, similar to the Malaysian population and, in a lesser extent, to the continental eastern populations (Fig. 1). On the other hand, Indonesian and continental populations are two divergent lineages discriminated by both mitochondrial and nuclear markers. Most of the similar patterns of melanisation found between these two lineages are thus convergences with different evolutionary origins.

In theory, such convergence could be either induced by a high phenotypic plasticity or by convergent evolutions of genes regulating the melanisation process. Phenotypic plasticity of the coloration has been shown in butterflies through day-length influence on the hormonal production [80] and in paper wasps through variation in rearing temperature [81] and diet [82]. Tibetts highlighted that diet influenced only limited melanin patterns involved in social signal such as the clypeus markings [82]. MacLean and her collaborators showed that colour variation in paper wasps induced by a rearing temperature change did not reach the inter-population variation [16]. Furthermore, diet of a single colony of hornet can change through the season in temperate areas [79] without inducing a colour variation in individuals (pers. obs.). Finally, in contrast to the paper wasps studied by Green *et al.* [81], hornet larvae develop in enclosed nests with buffered temperature and humidity, lowering the influence of external physical factors [83], [84]. Dark specimens of *V. velutina* from semi-tropical China have been accidentally introduced to temperate areas of France and Korea several years ago [52], [85] and these invasive populations did not change in coloration [85], [86]. It is therefore unlikely that the observed convergences in coloration of several *V. velutina* populations are due to phenotypic plasticity induced by similar local factors.

A more probable hypothesis is a parallelism in the evolution of the regulatory and patterning genes either by similar or dissimilar mutations [87]. This evolution may also have been influenced by extrinsic factors through selection: melanism, with a genetic basis, can have both positive and negative impacts on the organism's fitness [19], [20]. The different factors that may induce a selection on melanism are related to the climate and associated environments, but also to the local communities of predators and of Müllerian mimics.

### Climatic pressures

Climate is known to have an influence on melanism in insects. Melanism has been related to thermoregulation [88] and dessication resistance [89], [90]. Higher melanic insects were found to warm up faster under the sun, to reach higher temperature, but also to lose less water and resist desiccation better than lighter morphs [88], [89].

However, studies do not agree on the distribution of melanic forms. Some authors suggest that melanism occurs mostly in dry environments [89], while other argues that they are found in cool and wet habitats [16], [88] or in tropical areas [91]. Our results concur only partially with this last study. Two of the least melanised populations occur respectively in Nepal and in the driest island of the distribution of *V. velutina* in Indonesia while geographically intermediate populations are mostly darker, with the exception of the population from Malaysia. However, we found only low structured climatic differences between the localities of light and dark coloured populations and no cline is visible (Fig. 1). Melanism does not appear to be influenced by altitude, average or extreme temperatures or humidity of the locality of sampling. Especially, the difference between the geographically close Malaysian and Sumatran colour forms, respectively the lightest and the darkest, rejects the hypothesis of climate pressures as the major evolutionary force driving colour polymorphism in *V. velutina*.

### Predator pressures

Besides physical elements of the environment, hornets are likely to be selected for the efficiency of their warning colours [4]. Like other social wasps, hornets have a painful sting and are distasteful due to their venom gland, making them avoided by predators [8] [92], [93]. They benefit from being recognized by potential predators that may attack them or disturb their nests. Furthermore, predator avoidance may be critical in one part of the life-cycle of *V. velutina*: like in many other social wasps, each colony is annual and founded by one solitary queen [79]. At this time, selective pressures within the species are high, as each queen has to survive for weeks before being able to produce the next generation. As such, warning colours play an important role in social wasps by protecting queens during their solitary phase.

The notion of warning colours depends on the perception of colours by predators [94], [95]. While dark coloration may be advantageous for crypsis [19], the yellow coloration seems to be a warning colour naturally avoided by bird predators [9]. Vidal-Cordero and his collaborators also showed a direct link between the lightness of the metasoma pigmentation in a paper wasp and the size of its venom gland [96]. Light coloration may therefore intervene in predator avoidance through its intensity.

On another hand, black and yellow stripes patterns help in prey recognition both by vertebrate [97], [9] and invertebrate predators [8]. This may explain the presence of black and yellow metasomal stripes in most of the *V. velutina* populations and the potential patterns observed in mesosoma: the pronotum, the scutellum and sometimes the metanotum can become lightly coloured while the mesoscutum and the propodeum always present black markings. This coloration creates over the mesosoma an alternating pattern of light and black colours extending the striped pattern of the metasoma (Fig. 4.B). It is therefore unsurprising to find yellow marks even on the darkest morphs of *V. velutina* as well as black segments and markings on the lightest populations (Figs. 1 & 4).

### Müllerian mimicry

The influence of warning signal on predator behaviour depends on other harmful species encountered by the predators through the process of Müllerian mimicry, different harmful species sharing a similar aposematic signal, thus mimicking each other. This mimicry reinforces the impact of warning coloration by augmenting the probability of association between the bad experience of the predator and the signal displayed [4]. This phenomenon has been widely studied in butterflies [98], [12], but is also known among bumblebee species [91], [99], [100] and wasps [101]. Both being stinging Hymenoptera, bumblebees, bees and wasps are likely to be recognized as similar prey items by predators and may share the Müllerian effect of their coloration. For example, Hines and Williams pointed out drastic variation of colour in bumblebees from Malaysia, for which specimens are fully orange [100], matching the singular orange morph *divergens* of *V. velutina* found in the same region. Another example is the mimicry between the *nigrithorax* form of *V. velutina* and queens of two yellow jacket species *Vespula koreensis* (Radoszkowski, 1887) and *Vespula orbata* (du Buysson, 1902) found in southern China.

Presence of Müllerian mimicry is also often accompanied by strong phenotypic variation structured in mosaic across the distribution of the species [28]. Polymorphism between populations of *V. velutina* may thus be the result of different selective pressures induced both by variation in the local stinging Hymenoptera communities and by the pressures on melanism-related traits: crypsis, thermoregulation, desiccation and pathogen resistance. While hornets may not be highly sensitive to some of these pressures, co-mimic species with open nests or solitary habits may be, thus leading to an indirect effect of these factors on hornet coloration. Furthermore, the critical phase in colony development associated with the potential founder effects in island colonisation during *Vespa velutina* evolutionary history may explain the high variety of coloration found across islands while colour variation is more progressive and colour forms more widespread in continental Asia.

In order to test for this hypothesis, further studies could focus on characterising the melanism of the different species of stinging Hymenoptera of similar sizes in different regions. This work has already been started on bumblebees [91], [99], [100] but should be extended over the different families of Hymenoptera, as mimetism is not restrained taxonomically.

## Conclusion

Our study described the changes in patterns of melanisation over the distribution of the hornet species *Vespa velutina*. The main axis of pattern variation in the resulting colour space described a global melanisation of the body indicating that the melanisation is a partially integrated process across the distribution. Correlations among the melanisation of the different body parts revealed a structured variation with two apparent modules: one composed of the head capsule, anterior part of mesosoma and anterior legs and the other connecting melanisation of the anterior metasomal dorsum and metanotum.

Based on mitochondrial sequences and microsatellite markers, we identified two highly divergent lineages within the *V. velutina* species: a continental lineage and a lineage restricted to southern Indonesian islands. The existence of a continental lineage confirmed the synonymy of *V. auraria* under *V. velutina*. The evolutionary history of the species could not explain the observed colour variations: the variation in melanisation clearly included convergence in different populations. Comparison of the climatic, genetic, geographic and colour diversities showed that melanism was unlikely driven by abiotic factors such as climate variation or latitudinal clines. This variation may result instead of mutations selected by high local constrains on aposematism and Müllerian mimicry with potential influence of the founder effects in islands.

Together, these results confirmed that colour patterns should not be regarded as reliable criteria for population relationships or species distinction in social wasps. They also suggest that colour variation in aposematic species is not tightly related to abiotic conditions. Further studies could use the quantification of colour patterns to track correlated changes between species within communities of aposematic species and test for the influence of Müllerian mimicry, predators and habitat types on the geographic variation of aposematic coloration.

## Supporting Information

Appendix S1 **List of light colour characters with their modalities.** All modalities are depicted in Figure 4. The coding for the different modalities is mentioned in brackets for the 23 light coloration characters. The coding for the 23 dark characters is equivalent to one minus the corresponding light coloration character.
(DOC)
