# Supplementary material for: Geographic Variation of Melanisation Patterns in a Hornet Species: Genetic Differences, Climatic Pressures or Aposematic Constraints?
Source: PLoS One. 2014 Apr 16;9(4):e94162. doi: 10.1371/journal.pone.0094162 (PMC3989226; doi:10.1371/journal.pone.0094162)
Supplement: Appendix S1 — List of light colour characters with their modalities. All modalities are depicted in Figure 4. The coding for the different modalities is mentioned in brackets for the 23 light coloration characters. The coding for the 23 dark characters is equivalent to one minus the corresponding light coloration character. (DOC) [file pone.0094162.s001.doc]

**Appendix S1.**

Head (Figure 4.A)

01. Upper gena: (0) Black; (0.5) Brown or with a black spot; (1) Orange

02. Vertex: (0) Black; (0.5) Brown or with light marks; (1) Orange

03. Ocellar area: (0) Black; (0.5) Black spot; (1) Orange or Yellow

04. Dorsal margin of the scape: (0) Mainly black; (0.5) Intermediate; (1) Mainly orange

Mesosoma (Figure 4.B)

05. Prothorax: (0) Black; (0.33) Black with orange posterior margin; (0.67) Brown or Orange with black marks; (1) Entirely coloured dorsally.

06. Mesoscutum: (0) Entirely black; (0.25) Two faint latero-median brown bands; (0.5) Two thin latero-median orange/brown band; (0.75) Two wide latero-median band. (1) Extensively coloured.

07. Scutellum: (0) Black; (0.5) Colour marks; (1) Entirely brown or orange.

08. Metanotum: (0) Black; (0.33) Orange spots on each side; (0.67) Orange bands not reaching the middle; (1) Orange.

Mesosoma (Figure 4.C)

09. Mesepisternum: (0) Entirely black; (1) Yellow spot in the upper part.

Legs (Figure 4.C)

10. Profemora: (0) Black; (0.5) Brown with black marks; (1) Orange.

11. Protibia: (0) Black; (0.5) Brown with black marks; (1) Orange.

12. Metatibia apex: (0) Black; (1) Black with a light spot.

Dorsal metasoma (Figure 4D)

13. 1st metasomal tergum: (0) Black; (0.33) Black with brown marks; (0.67) Brown with an intermediate black line; (1) Widely orange.

14. Basal area of the2nd metasomal tergum: (0) Black; (1) Brown or orange marks.

15. Apical margin of the 2nd metasomal tergum: (0) Black; (0.5) Wide apical orange margin; (1) Orange.

16. 3rd metasomal tergum: (0) Black; (0.33) Sub-rectangular black mark; (0.67) Medial black mark; (1) Orange.

17. 4th metasomal tergum: (0) Black; (0.33) Black rectangular medial mark; (0.67) Black triangular medial mark; (1) Orange.

18. 5th metasomal tergum: (0) Black; (0.5) Brown or medial black mark; (1) Orange.

19. 6th metasomal tergum: (0) Black; (1) Brown or orange.

Ventral metasoma (Figure 4.E)

20. 2nd metasomal sternum: (0) Black; (0.33) Pair of basal spots or apical brown mark; (0.67) Basal and apical orange mark; (1) Extensively orange.

21. 3rd metasomal sternum: (0) Black or brown; (0.5) Middle black marking; (1) Orange.

22. 4th metasomal sternum: (0) Black; (0.5) Mainly brown; (1) Orange

23. 5th & 6th metasomal sterna: (0) Black; (1) Other colour.
